# Synthesis and Characterization of Green Carbon Dots for Scavenging Radical Oxygen Species in Aqueous and Oil Samples

**DOI:** 10.3390/antiox9111147

**Published:** 2020-11-19

**Authors:** Clarissa Murru, Rosana Badía-Laíño, Marta Elena Díaz-García

**Affiliations:** Department of Physical and Analytical Chemistry, Faculty of Chemistry, University of Oviedo, 33006 Oviedo, Spain; murruclarissa@uniovi.es (C.M.); rbadia@uniovi.es (R.B.-L.)

**Keywords:** carbon dots, green synthesis, green antioxidants, luminescent nanoparticles, antioxidant activity, water and oil solubility

## Abstract

Carbon dots (CDs) due to their unique optical features, chemical stability and low environmental hazard are applied in different fields such as metal ion sensing, photo-catalysis, bio-imaging and tribology, among others. The aims of the present research were to obtain CDs from vegetable wastes (tea and grapes) as carbon sources and to explore their potential properties as radical scavengers. CDs from glutathione/citric acid (GCDs) were synthetized for comparison purposes. The CDs were investigated for their chemical structure, morphology, optical and electronical properties. The antioxidant activity has been explored by DPPH and Folin-Ciocelteau assays in aqueous media. Due to their solubility in oil, the CDs prepared from tea wastes and GCDs were assayed as antioxidants in a mineral oil lubricant by potentiometric determination of the peroxide value. CDs from tea wastes and GCDs exhibited good antioxidant properties both in aqueous and oil media. Possible mechanisms, such as C-addition to double bonds, H-abstraction and SOMO-CDs conduction band interaction, were proposed for the CDs radical scavenging activity. CDs from natural sources open new application pathways as antioxidant green additives.

## 1. Introduction

The emergence of carbon dots (CDs) has attracted much attention due to their unique luminescent properties, their chemical stability, their low toxicity and low environmental hazard. A broad range of technological applications such as optical sensing [1], bioimaging [2], tribology [3] or photocatalysis [4], among others, have been extensively explored. Furthermore, due to their ability for scavenging reactive oxygen species, CDs demonstrate significant potential, particularly for biological applications [5,6,7]. CDs can be prepared from different natural resources such as albumin, chitosan, gelatin, peels of fruits, grains or other plant matters as well from vegetable wastes (e.g., apple or grape pomace), which make them “green” nanoparticles, as described in several excellent reviews devoted to the synthesis of CDs from green biomass wastes, their surface modification to improve their physico-chemical properties and their applications in bioimaging, biosensing, catalysis, forensics and optoelectronics [8,9,10,11,12]. The study of CDs as antioxidant nanoparticles is still in its infancy and some examples are exemplified in Table 1, in which it could be observed that most of precursors used to produce CDs with antioxidant properties are vegetal-based materials. Only a few of such CDs have been applied to real samples, particularly to in vitro evaluate cells viability and protection under oxidative stress. Besides, the method commonly used to prepare these CDs is the hydrothermal (HT) synthesis. 

Research efforts have yet to be made to utilize such scavenging ability in other fields such as food industry (e.g., packing films), cosmetics, pharmacy, industry and lubrication. In the particular case of oil lubricants (mineral, synthetic or vegetal-based), one of the degradation processes common to them is their reaction with atmospheric oxygen, not only during their fabrication or storage but particularly during use. Once the lube is oxidized, the quality requirements are no longer met, which results in economic and energy losses as well as possible machinery failure owing to an increase of friction and wear between the moving surfaces. Eco-friendly additives are being developed to improve the low oxidative stability of lubes, particularly of biodegradable vegetal-based lubricants due to their unsaturated fatty acid composition [26]. 

The aim of the present work was to develop CDs using vegetable wastes (tea and grape pomaces) as carbon source and glutathione/citric acid as comparative CDs model. The CDs were systematically investigated in detail over their morphology, spectroscopic properties and electronic properties. Besides, their antioxidant activity has been explored by DPPH and Folin-Coicelteau assays in aqueous media. The solubility of these CDs in oily media was explained by the presence of reverse micelles and, as proof of the concept, the CDs prepared from tea wastes were assayed as green antioxidant additives in a mineral base oil, for the first time. Results showed that the CDs from tea wastes have good antioxidant properties in oil media when compared with CDs from grape pomace. The possible radical scavenging activity mechanisms were outlined for both aqueous and oil media. These results, along with the unique optical properties of CDs, excellent photostability, low toxicity and environmental friendliness [27] may open a way for designing new luminescent CDs with improved antioxidant properties for biomedical, bioimaging, chemical and industrial applications.

## 2. Materials and Instrumentation

### 2.1. Chemicals

The base oil (ISO-68) employed in this study was kindly supplied by REPSOL (Madrid, Spain). Tea leaves were purchased from local markets. Glutathione, citric acid, gallic acid, 2,2-diphenyl-1-picrylhydrazyl (DPPH reagent), chloroform, glacial acetic acid, potassium iodide and sodium thiosulfate were obtained from Sigma-Aldrich Co. (Barcelona, Spain). Folin-Ciocalteau’s phenol reagent was purchased from Merck (Madrid, Spain). The grape pomace was supplied by the Bodegas Vidas winery (Cangas de Narcea, Asturias, Spain). Green tea bags of the brand Hacendado were obtained from a local supermarket (Mercadona). Deionized Millipore water (MQW) was used throughout the experimental work.

### 2.2. Instrumentation

Carbon dots samples were characterized using UV–visible spectroscopy with a Lambda 900 UV/VIS/NIR spectrophotometer (Perkin Elmer Spain, Madrid, Spain). Photoluminescence emission spectra were recorded on a Varian Cary Eclipse spectrofluorimeter from Agilent Technologies (Las Rozas, Madrid, Spain), equipped with a xenon flash lamp. Absorption and photoluminescence spectra were registered using 1-cm quartz cells. Luminescence quantum yields (QY) were measured directly by absolute measurement system on a FS5 fluorometer (Edinburgh Instruments, Livingston, UK) equipped with an integrating sphere module and the calculation was performed by the instrument’s FluOracle^®^ software. Morphological details and size determination of CDs were determined by using a High Resolution Transmission Electron Microscope (JEM-2100F, 200 kV, JEOL, Fresing, Germany). The average diameter and the standard deviation were determined from digitalized images using a ImageJ Tool software. Attenuated total reflectance Fourier Transform Infrared Spectroscopy (ATR-FTIR) spectra were collected on a Varian 670-IR spectrometer (Varian, Madrid, Spain) equipped with a Golden Gate ATR device. The spectra were scanned between 600 and 4000 cm^−1^. The spectral resolution was 4 cm^−1^ at 2000 cm^−1^ as the average of 32 scans. Background was collected with the ATR crystal exposed to air.

### 2.3. Synthesis of Carbon Dots

#### 2.3.1. Glutathione/Citric Acid CDs

Gluthathione-based CDs (GCDs) were synthesized by a hydrothermal method as described by Díaz-Faez et al. [28] with some modifications: 1 g of glutathione and 1 g of citric acid were thoroughly mixed in a silica crucible and then 10 mL of MQW were added to form a clear solution that was then heated in an oven for 5 h at 180 °C. During the heating process, several aliquots of MQW (1 mL water every 30 min) were added to avoid the product to scorch. Once cooled at room temperature an orange-brownish gel was obtained which was then dissolved in 10 mL MQW. The obtained CDs were purified by dialysis using a tube membrane (MWCO, 3.5 kDa) against MQW during 24 h. The dialyzed solution was freeze-dried by a vacuum freeze dryer. Finally, the glutathione-based CDs obtained were stored at 2 °C for further use. The synthesis yield resulted to be 5.4%. Stock dispersions in Milli-Q water containing 0.54 mg mL^−1^ were prepared for further characterization and use. Citric acid/glutathione based CDs were taken as reference CDs along this work for two reasons: i) the presence of N and S heteroatoms (provided by glutathione) that improve their photoluminescence properties (e.g., high quantum yield) [28,29] and ii) due to the GCDs demonstrated antioxidant activity [30]. The synthesis was performed in triplicate.

#### 2.3.2. Tea Waste-Based CDs

The tea-based CDs were synthesized by a carbonization method using the waste green tea leaves as a carbon source. After preparing green tea infusions, the contents of three bags were air dried at room temperature and then grounded into a fine powder. Two g of ground tea powder was transferred into a Teflon beaker and heated at 200 °C for 6 h in an oven. Once cooled at room temperature, the black powder obtained was dispersed in MQW and gently stirred overnight. The mixture was filtered through 100 µm filter paper and centrifuged at 12,000 rpm to remove larger particles. The supernatant liquid was filtered by a 0.22 μm microfiltration membrane and the solution was purified by dialysis using a tube membrane (MWCO, 3.5 kDa) against MQW during 24 h. Finally, the concentrated solution was freeze-dried by a vacuum freeze-dryer. The obtained CDs are hereafter named as TCDs and were stored at 2 °C until further use. The synthesis yield resulted to be 5%. Stock dispersions in Milli-Q water containing 0.52 mg·mL^−1^ were prepared for further characterization and use. The synthesis was done in triplicate.

#### 2.3.3. Grape Pomace-Based CDs

For the synthesis of CDs from grape pomace, two different routes were addressed. In the first case (hydrothermal-assisted process), 2 g of grape pomace were added to 20 mL of 1 M NaOH solution. The mixture was transferred into a 50 mL Teflon-lined stainless-steel autoclave and heated to 180 °C for 4 h in an oven. Once the mixture was cooled at room temperature, the reaction product was centrifuged at 5000 rpm for 15 min to remove any precipitate. The supernatant was dialyzed against MQW through a dialysis bag for 4 days. Finally, the purified solution was freeze-dried and the CDs (named as P1CDs) stored until further use. The synthesis yield resulted to be 6%. In the second process (open vessel) 2 g pomace were transferred into Teflon beaker and heated at 180 °C for 4 h in an oven. Every 30 min 1 mL MQW was added to avoid complete carbonization. The reaction product was suspended in MQW, centrifuged and dialyzed as described above. The solution was freeze-dried and the CDs (named as P2CDs) stored at 2 °C until further use. The synthesis yield resulted to be 2.5%. Stock dispersions in Milli-Q water of P1CDs 0.56 mg mL^−1^ and P2CDs 0.58 mg mL^−1^ were prepared for further characterization and use. The syntheses were performed in triplicate.

### 2.4. Measurement of Total Reducing Capacity: Folin-Ciocalteau Procedure

The Folin-Ciocalteau (FC) method, known also as the total phenolic assay, provides the reducing capacity of a material and it is usually expressed as phenolic content. External calibration was done using different concentrations of gallic acid. In brief, 250 μL of working dispersions of the different CDs were mixed with 100 µL of FC reagent (mixture of phosphotungstic acid, H_3_PW_12_O_40_ and phosphomolybdic acid, H_3_PMo_12_O_40_), 250 µL Na_2_CO_3_ 7.5% *w/v* and MQW up to a final volume of 10 mL. The mixture was gently mixed and after 30 min reaction at room temperature in the dark, absorbance was measured at 765 nm against a regent blank. Measurements were carried out in triplicate and data presented as the average. The reducing capacity was calculated as mg of gallic acid equivalents per mL by using a gallic acid calibration curve.

### 2.5. DPPH Free Radical Scavenging Assay

The DPPH free radical scavenging assay was carried out according to the following protocol: 1 mL of ethanolic solution of DPPH (100 µM) was added to a given volume of the different CDs dispersions. The mixture was shaken and after standing for 30 min in the dark, the absorbance at 517 nm was measured to determine the concentration of the remaining free DPPH^•^ radical. All tests were performed in triplicate and MQW was used as a blank control. The scavenging efficiency of the three kinds of CDs for DPPH^•^ was calculated using the Equation (1):

DPPH inhibition % = [(A_c_−A_s_) × 100/A_c_] (1)

where A_c_ is the absorbance of the control and A_s_ is the absorbance of the sample.

### 2.6. Determination of the Lubricant Oil Peroxide Value

The peroxide value (PV) is a measure of the degree of peroxidation and allows to determine the amount of total peroxides which oxidizes potassium iodide. The iodine released by the peroxides is then volumetrically determined by a standard solution of sodium thiosulfate and the equivalence point determined potentiometrically using a combined Pt (sensing ring) and Ag/AgCl 3 M KCl, 3M KNO_3_ reference electrode. A pH/ion-meter GLP 22 (Hach Lange Spain, S.L.U., Barcelona, Spain) with an accuracy ±0.2 mV was used for redox potential measurements. The thiosulfate solution was added using a micropipette. The PV in presence of CDs as antioxidant additives was performed according the following procedure: three oil samples were prepared, two containing 0.25% *w/v* of carbon dots (GCDs or TCDs) and one without CDs was used as blank. The samples were heated during 15 days with moderate stirring under oxygen atmosphere and raising the temperature to 75 °C to allow a slow formation of reactive chemical species. To evaluate the PV, 10 g of each sample were weighed and dissolved in 50 mL of a mixture of Cl_3_CH/glacial acetic acid (3:2 *v/v*). Then, 0.5 mL of saturated solution of KI aqueous solution was added. The mixture was gently stirred up to the finish of the analysis. The Pt-Ag/AgCl combined electrode was immersed into the two-phase system and stable redox-potentials were measured after each addition of 0.005M sodium thiosulfate. Similar operations were performed for the blank solution. The PV was calculated using Equation (2):

(2) $$PV=\frac{\left(V-V_{0}\right)\;\times \;C_{t}\;\times F\times 1000}{m}$$

where *V* and *V*_0_ are the volumes of the sodium thiosulfate standard solution additions to the analyzed lubricant oil sample and to the blank, respectively. *F* is the correction factor of the sodium thiosulfate solution. *C_t_* is the molar concentration of the standard solution of sodium thiosulfate and m is the weight (g) of the lubricant oil sample.

## 3. Results and Discussion

### 3.1. Morphological Characterization

**Figure 1:**
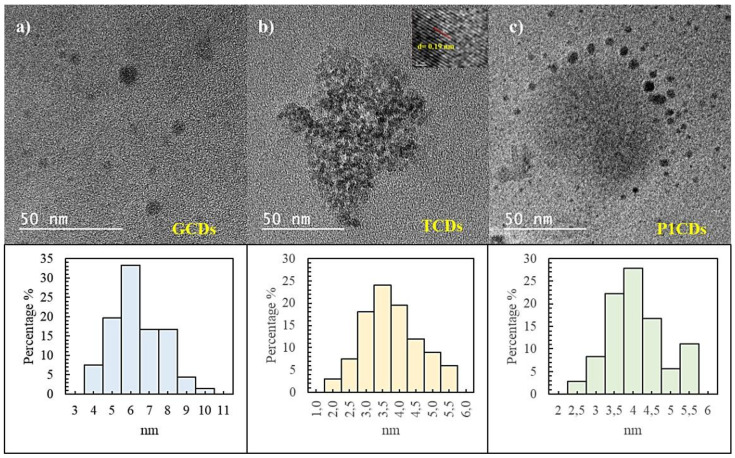
HR-TEM images of (**a**) GCDs, (**b**) TCDs and (**c**) P1CDs samples and their corresponding size distribution histograms; *n* = 118 TCDs, *n* = 106 GCDs, *n* = 144 P1CDs.

All the as-prepared CDs were highly water soluble as well as in ethanol. The particle size and morphology of the CDs were characterized by HRTEM. As can be seen in Figure 1a the GCDs are mostly monodispersed with an average diameter size of 6 ± 0.8 nm, while TCDs are smaller with an average diameter size of 3.5 ± 0.6 nm (Figure 1b). Also the inset HRTEM image in Figure 1b clearly shows the presence of both an amorphous phase and a crystalline graphite phase with a lattice spacing of 0.19 nm, that closely matches with that of in-plane lattice spacing (010) of graphene (d_100_ = 0.213 nm) [31,32] and points out the crystallinity of these TCDs. The P1CDs (Figure 1c) and P2CDs (Figure S1) are also monodisperse with particle diameters between 3 and 5 nm, with an average size of 4 ± 0.8 nm (Figure 1c).

### 3.2. Characterization of Functional Groups

**Figure 2:**
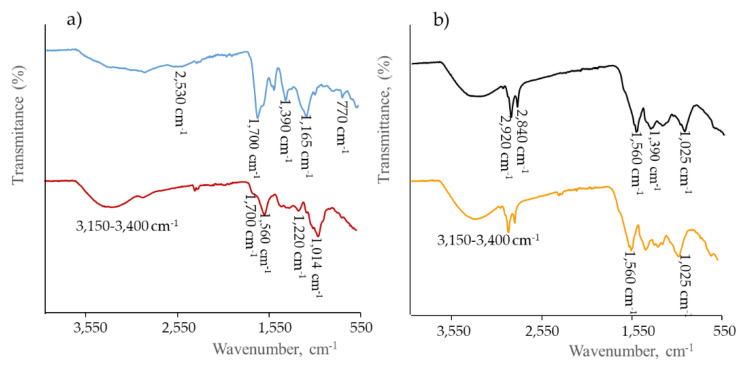
FTIR spectra comparison of: (**a**) GCDs (**—**) and TCDs (**—**); (**b**) P1CDs (**—**) and P2CDs (**—**).

The chemical structure of the CDs was also characterized by ATR-FTIR analysis. The broad bands at 3150–3400 cm^−1^ in all CDs samples were ascribed to -OH stretching and N-H bonds stretching (Figure 2). In the case of GCDs the transitions displayed at 1700, 1165 and 1070 cm^−1^ were assigned to ester COO– vibrations. Also, other groups were identified: amide (-ONH-, 1652 cm^−1^), carbonyl (-C=O, 1695 and 1322 cm^−1^), amine (combination of C–N stretching band and N–H bending at 1540 cm^−1^), CH_3_ out of plane bending (1390 cm^−1^) and stretching vibration bands due to S–H and C–S bonds at 2530 cm^−1^ and 771 cm^−1^, respectively. 

P1CDs and P2CDs spectra show a broad peak from 3600 cm^−1^ to 3200 cm^−1^ which relates to the O-H and N-H bonds stretching present in lignocellulose components of the grape pomace [33]. The peaks around 2840 cm^−1^ and 2920 cm^−1^, also observed in the TCDs spectra, were attributed to symmetric and asymmetric stretching of C-H bonds in methyl and methylene groups. The band at 1560 cm^−1^ attributed to aromatic C=C vibrations was also observed in both P1CDs and P2CDs spectra, as well as the band at 1390 cm^−1^ due to CH_3_ out of plane bending attributed to polysaccharide structure. The band at 1220 cm^−1^ was assigned to alkyl aril ethers (-OCH_3_) and the band at 1025 cm^−1^ was attributed to vibrations of C-6 of cellulose [34]. In the case of TCDs, the peak at 1600 cm^−1^ was ascribed to the vibration of the amide I (N-H bending), the peak at 1014 cm^−1^ to C-O groups in polysaccharides and the peak at 1700 cm^−1^ to simple ketones and carboxylic groups. It is worth mentioning that some of these bands, due to the composition of the starting materials to prepare the CDs, may be overlapped by the bands of graphene/graphite generated during their synthesis. For example, for graphene oxide the IR strong broad-band centered at ~3430 cm^−1^ has been assigned O–H stretching vibrations, the peaks around 2925 and 2845 cm^−1^ attributed to sp^2^ and sp^3^ C-H stretching bands, the peak at 1620 cm^−1^ assigned to the aromatic C=C stretching, that at about 1400 cm^−1^ to the O–H deformation and the 1260 cm^−1^ peak to C–OH stretching [35,36]. 

### 3.3. Spectrophotometric and Luminescent Optical Properties

**Figure 3:**
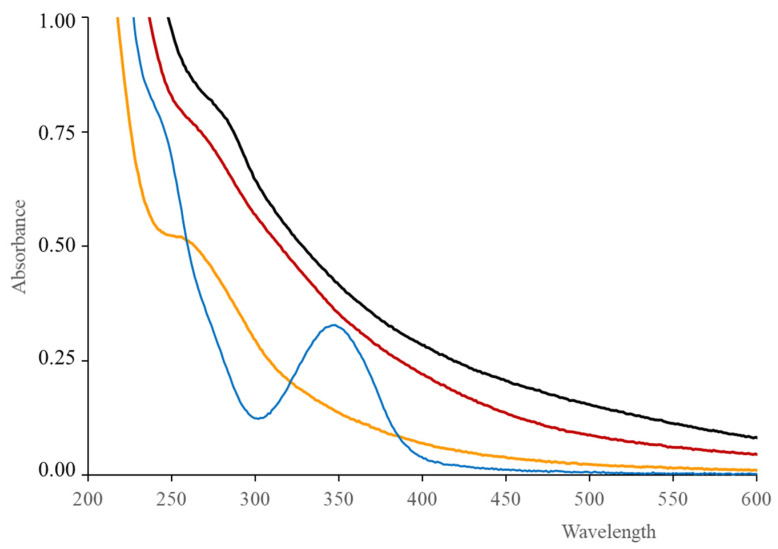
Optical absorption spectra of GCDs (**—**), TCDs (**—**), P1CDs (**—**) and P2CDs (**—**) in aqueous solution.

The study of the optical properties of the CDs was carried out in detail. In Figure 3, the UV–Vis absorption spectrum of GCDs shows a typical absorption band at 347 nm ascribed to n–π* transitions (C=O bonds) and a shoulder around 240 nm attributed to π-π* transitions (C–C bonds with sp^2^ hybridization). This spectrum differs from those of the other CDs for which the peak at about 350 nm was not observed. However, compared with that of GCDs, a slight red shifting was observed for the 240 nm absorption shoulder: to 258 nm in P1CDs, to 265 nm in TCDs and to 275 nm in P2CDs, which may indicate that this band, or in turn, the relative amount of sp^2^ C, are affected by the chemical composition of the CDs.

**Figure 4:**
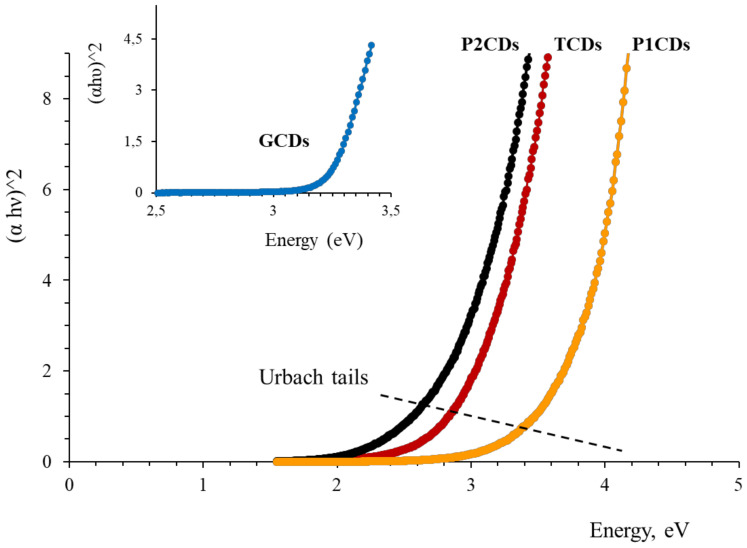
Tauc plots for estimation of the CDs bandgap.

From the analysis of the absorption spectra, the bandgap (*E_g_*) for the different CDs was estimated using the Tauc’s law for direct transitions [37] (Equation (3)):

(αhν)^2^ = k (hν−E_g_) (3)

where *α* is the absorption coefficient, hν the energy of the incident light and k is a constant. E_g_ was estimated from the graphical representation of (ahν)^2^ vs hν by extrapolating the straight line to (ahν)^2^ = 0 (Figure 4). The E_g_ values are recorded in Table 2.

The absorption edge of the CDs was not abrupt and an exponential zone, called Urbach tail, can be observed below the optical bandgap (Figure 4). These tails have been ascribed, to disorders, defects and thermal vibrations in the lattice of the material [38,39]. Urbach tails are associated with absorption bands of localized electronic centers in semiconductor crystals. The absorption coefficient near the band edge depends in an exponential manner on the photon energy and obeys the Equation (4):

α(hν) = α_0_ exp (hν/E_u_) (4)

where *α_0_* is a constant and E_u_ is the Urbach energy (see Supplementary Material). The value of E_u_ can be estimated by taking the reciprocal of the slopes of the linear segment in the lower photon energy region of the ln(α) vs hν curve. With the aim to evaluate the structural quality of the synthesized CDs, E_u_ were estimated using the above relation (Figures S2–S5). Results are collected in Table 2. The small value of E_u_ for GCDs indicates that these nanoparticles exhibit a nanostructure with less defects than the rest. TCDs, with the highest E_u_, may have a nanostructure more amorphous and richer in defects. 

### 3.4. Luminescence Properties

In Figure S6 the excitation and emission spectra of the synthetized CDs are shown. GCDs exhibit an intense emission band centered at 418 nm upon excitation at 350 nm. Taking the photoluminescence spectra of GCDs as reference, we can observe that while the maximum excitation wavelength of P1CDs remained at ca. 350 nm the maximum emission slightly shifted to 432 nm for P1CDs. In Table 2 the spectral characteristics of all the synthetized CDs and the spectral shifts respect to the GCDs are collected. Fluorescence spectra of TCDs and P2CDs are shown in Figure S6. Also, in comparison with the spectral characteristics of GCDs, the maximum excitation of P2CDs shifted to the blue (318 nm) and the maximum emission wavelength was at 440 nm with a very weak fluorescence intensity. The synthesis procedure of P2CDs must be behind the poor photoluminescent behaviour of these nanoparticles, as proposed in different works [40,41]. Begheri et al. [42] have suggested that during the thermal treatment, along with CDs more carbonized carbon particles (MCCPs) may also be formed as a major by-product, which have quenching effects on the photoluminescence of the CDs. The full width at half maximum (FWHM) of the emission band was used to evaluate both the homogeneity of the CDs [43] and the synthesis reproducibility. So, the fluorescence emission band of GCDs with its maximum intensity at 418 nm presented a FWHM of (126 ± 4.5) nm. In the case of TCDs, the maximum emission wavelength appeared at 430 nm and the band has a FWHM of (116 ± 5.4) nm and, finally, the emission band of P1CDs was accompanied by a broader FWHM of (157 ± 8) nm and an emission maximum at 423 nm. The low standard deviations observed in the FWHM indicate a high reproducibility of the corresponding synthesis. 

The quantum yield (QY) value is a measure of the quality of the synthesized CDs. The absolute QY of all CDs fabricated are collected in Table 2. The highest QY is observed for GCDs while those of the rest of CDs are clearly lower. These findings correlate to the presence of the C=O, C-OH and nitrogen groups in TCDs, P1CDs and P2CDs (see FTIR analysis) which introduce surface and/or core traps, formation of dangling bonds and increased structural disorder (increase in *E_u_* energies), all of which may reduce the probability of radiative transitions (low QYs). Besides, the low QY for the P2CDs compared with that of P1CDs could be explained by higher amounts of –COOH groups at the edge of sp^2^-conjugated π-domains of P2CDs, owing to their synthesis was performed in the absence of NaOH. The reduction of -COOH groups (and their conversion to -COONa) resulted in a reduction of non-radiative electronic transitions which, in turn, enhanced the quantum yield [44]. On the other hand, the glutathione precursor used to create the GCDs introduced nitrogen and sulphur groups (see FTIR spectra in Figure 2), thus acting as an effective surface passivating agent [45]: the amount of disorder could be reduced (low band tail structural disorder E_u_), S-H and C-S bonding defect states in the band gap were created and radiative transitions significantly increased [46], thus GCDs exhibiting a comparatively high QY.

### 3.5. pH Surface States Influence on Luminescence Properties

**Figure 5:**
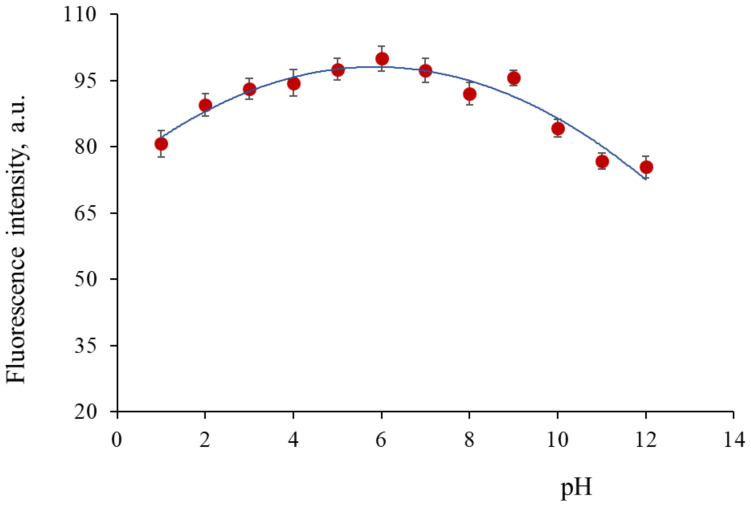
Fluorescence intensity changes upon pH variation in P1CDs.

In order to investigate the surface state effects (functional groups, particularly amine and carboxylic) on the photoluminescence properties of the CDs, the luminescence spectra were recorded within the pH range 1 to 12, using different buffer solutions: pH 2–4: (0.1 M glycine in 0.1M NaCl) + (0.1 M HCl). pH 5–8: 0.1 M KH_2_PO_3_) + (0.1 M Na_2_HPO_3_) and pH 8–12: (0.1 M glycine in 0.1 M NaCl) + (0.1 M NaOH). The final ionic strength of the measured solutions ranged from 0.32 to 0.36 M, so that its contribution to the photoluminescence of the CDs was expected to be marginal. Taking P1CDs as an example, Figure 5 shows that the fluorescence intensity did not show any significant change as the pH changed from 4 to 8 and then it decreased sharply from pH 8 to pH 12. A similar behavior has been observed for the rest of CDs. Below pH 4, the amino groups on the surface were easily protonated [47] and a slight decrease of the P1CDs emission was observed. At pH > 8, deprotonation of surface groups (amino N, C=O) was responsible for the decrease of the emission intensity. These observations indicated the fluorescence intensity depended on the surface state of the CDs. In the near/neutral pH range, the emission was practically constant, probably due to isoelectric situation among the surface groups. Furthermore, it was observed that the position of the emission peak centered at 423 nm did not change with the pH change. In the particular case of GCDs, the influence of pH was similar to that observed in our previous work [28]: within pH 3–8 the GCDs fluorescence smoothly increased and then decreased drastically as result of ionization of the functional groups present on the GCDs. 

### 3.6. Spectrophotometric Study of Antioxidant Activity

**Figure 6:**
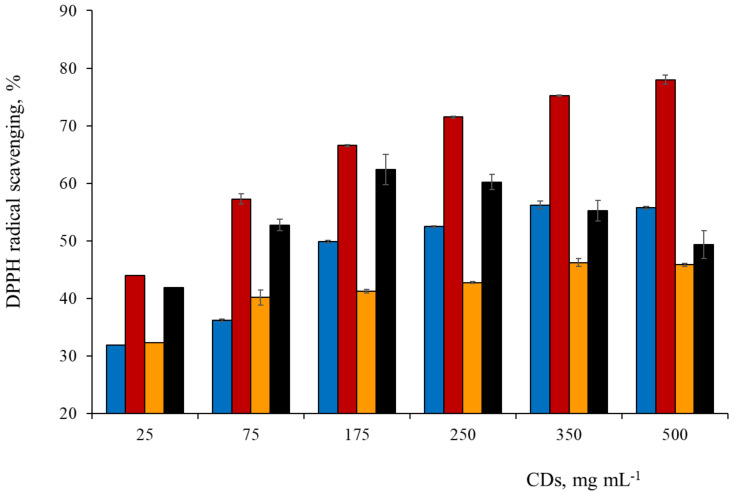
Antioxidant activity of the CDs at different concentrations: TCDs (■), GCDs (■), P1CDs (■) and P2CDs (■).

The Folin-Ciocalteu assay measures the total antioxidant capacity expressed as total phenolic content (TPC), although it does not necessarily reflect the total amount of phenols. The Folin-Ciocalteau method is a colorimetric electron transfer assay in which the redox reactions can take place not only with phenolic compounds but also with other compounds like reducing sugars or ascorbic acid [48]. The TPC of the different CDs was expressed as gallic acid equivalent (GAE), i.e., mg gallic acid mL^−1^, and results showed that P2CDs had the highest antioxidant capacity (0.17 mg mL^−1^ GAE), followed by TCDs (0.12 mg·mL^−1^) which may due to the high content of phenolic compounds (phenolic acids, flavan-3-ols, flavonols) present in the precursor materials. On the contrary GCDs and P1CDs were the samples with the lowest antioxidant capacity with 0.03 and 0.02 mg mL^−1^ GAE, respectively. The antioxidant activity was measured using the DPPH radical scavenging colorimetric assay based on the radical scavenging activity of antioxidants against the stable DPPH^•^ radical in methanol. When the free radical DPPH^•^ is reduced to hydrazine (a stable diamagnetic molecule) a color change is observed from purple (DPPH^•^ absorption at 517 nm) to yellow (hydrazine). The percentage scavenging effect of DPPH^•^ versus concentration of CDs were plotted in Figure 6. It was found that the DPPH activity of CDs was dose dependent. At 375 µg·mL^−1^ CDs concentration, the DPPH radical scavenging activity of TCDs, GCDs and P1CDs resulted to be 75%, 56% and 46%, respectively. The trend observed for P2CDs was different: there was an increase of the DPPH activity up to a concentration of 180 µg·mL^−1^ (62 %) and then slowly decreased to 49% at a concentration of 500 µg·mL^−1^. 

The correlation of TPC with antioxidant activity showed that TCDs and P2CDs with a high TPC had also a high DPPH radical scavenging activity in comparison with P1CDs with the lowest TPC. On the other hand, the average effective scavenger concentration (EC_50_) measurement was calculated in order to recognize the necessary amount of CDs to reduce 50% of the initial DPPH. The EC_50_ values were taken in the range of linear relationship between CDs concentration and DPPH inhibition percentage in order to avoid errors associated to the plateau or decreasing regions. The EC_50_ values obtained were 50 μg·mL^−1^ for TCDs, 75 μg·mL^−1^ for P2CDs, 175 μg·mL^−1^ for GCDs while P1CDs barely reached a 45% inhibition. These results clearly indicated that TCDs have a greater antioxidant activity than the pomace derived CDs or the GCDs.

Although there is scarce information about antioxidant activity of CDs obtained from crop wastes, the EC_50_ values of TCDs and P2CDs compare favorably with those for CDs obtained from coconut wastes and those of essential oils with antioxidant capacity. (see Table 3) using the DPPH assay. Pure ascorbic acid and vitamin E have the lowest EC_50_ values. 

**Figure 7:**
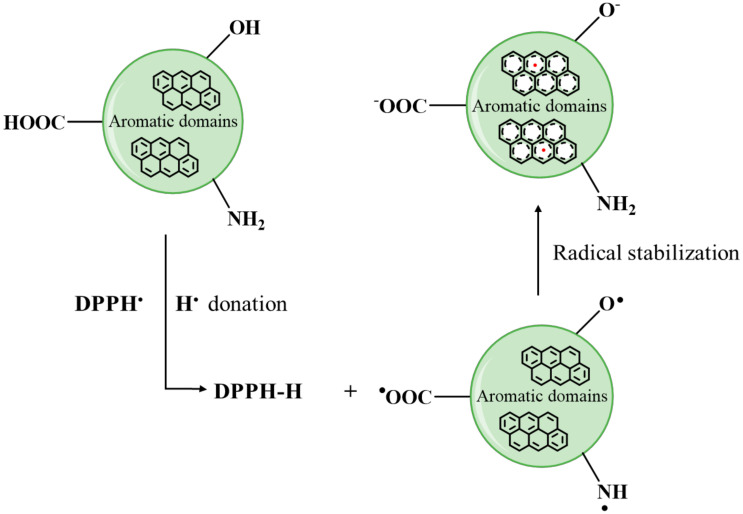
Mechanism of DPPH^•^ reduction by antioxidant CDs in aqueous media.

Considering all the results together, a possible mechanism for the DPPH^•^ reduction by the CDs could be based on the DPPH^•^ quenching by hydrogen atom transfer from the carboxyl, hydroxyl and/or amino groups as described in Figure 7 for TCDs, P1CDs and P2CDs. In the case of GCDs, the presence of –SH groups also has to be considered. DPPH^•^ is reduced to DPPH-H by taking up a H^•^ donated by any of the surface groups and the resultant unpaired electrons in CDs may be then delocalized by resonance within their aromatic domains or by rearrangement of chemical bonds.

### 3.7. Application of Carbon Dots as Antioxidant Additives in Non-Aqueous Media

**Figure 8:**
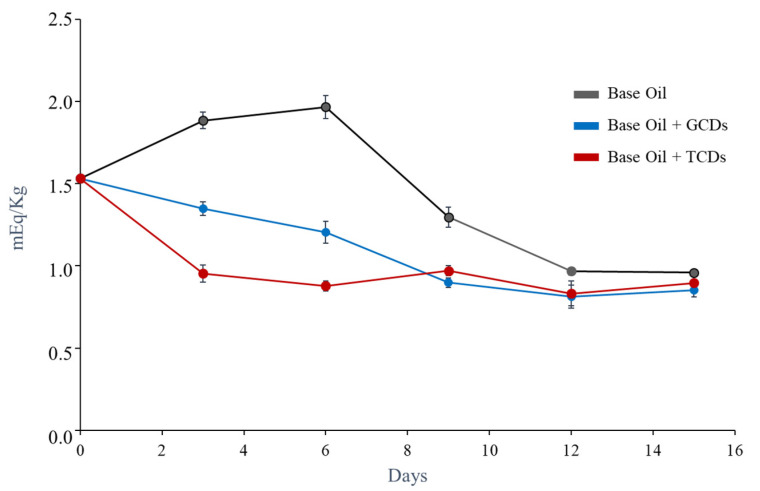
Study of the aging of lubricating oil samples with and without CDs additives.

Mineral oils are the most commonly used lubricants in industry and they differ from each other depending on the source of crude oil and the refining process. The fundamental differences between mineral oils are based on their chemical composition, sulfur content and viscosity. The inevitable oxidation of oils during their use results in undesirable increases in friction and wear, energy dissipation as heat and loss of efficiency in the mechanical system in which the lubricant oil is used. The oxidation of hydrocarbon oils is a radical chain reaction that may be initiated by the presence of oxygen, moisture, heat and metals that may accelerate the breakdown process [54]. During the oxidation process, hydroperoxide radicals are formed, and over the years different methods have been developed to determine the degree of oil oxidation. In particular, the peroxide value (PV) is one of the primary indicators used to assess the hydroperoxides formation and several methods are available to determine it. As a proof of the concept, and taking the advantage of the solubility of the as-synthetized CDs in oil, in this work TCDs and GCDs were assayed as possible antioxidant (radical scavengers) lubricant additives in the base oil ISO 68 (see properties in Table S1). In this work, PV was measured by the iodometric titration method with potentiometric measurement of the end-point as described in the Experimental Section. According the ISO official method [55] the sample size has to be adequately taken: for samples that are expected to have an amount of peroxide between 1 and 30 mEqO_2·_Kg^−1^ a weigh of 5 g with an approximation of 0.1 mg is recommended, while for samples that have a quantity less than 1 mEqO_2·_Kg^−1^ a weight of 10 g with an approximation of 0.1 mg should be taken. Results were plotted in Figure 8 and it can be observed that the most significant changes in the PV occur during the first days of the experiment. 

In absence of CDs an increase of the PV took place within the six first days due to the oil oxidation and then a sudden decrease was observed due probably to termination of the free radical chain reactions. The samples containing CDs showed more oxidative stability, being those containing TCDs the more stable since the first two hours. TCDs and GCDs offer potential as antioxidant additives in lubricant oils. Besides, due to the increasing concern over the environmental issues, the use of environmentally acceptable materials is mandatory.

Several works have demonstrated that the use of natural resources (cereals, seeds, aromatic plants, essential oils, etc) or renewable biomass make the development of C-dots more sustainable than the use of common organic molecules under the same experimental conditions [56]. In recent papers by Pinto da Silva et al. [57,58], it was demonstrated that the fabrication of CDs from standard organic precursors (citric acid as carbon source and urea or ethylenediamine (EDA) as N-doping molecule) can be performed environmentally sustainable by adequate selection of the synthesis route (e.g., microwave-assisted hydrothermal treatment) and the N-doping substance (EDA in this particular case). 

We should like also to mention that these CDs could also be used as green anti-friction and anti-wear additives in lubricant oils, showing a reduction of the friction coefficient of a 36% and a wear reduction of 10% respect to the neat base oil [59,60]. The solubility of the CDs in mineral oil deserves a brief discussion. The solubility of the hydrophilic CDs in the oil is closely related with the moisture present in the oil. The concentration of water may vary from 300 to 400 ppm for mineral oils, while for synthetic biodegradable oils it ranges from 800 to 1000 ppm [61]. Taking into account that the mineral oil ISO 68 was not previously dried in our experiments it should content, at least, the lowest level of moisture related with the room air humidity. This dissolved moisture is the driving force to form reverse micelles: amphiphilic oil components may self-assemble into roughly spherical structures (reverse micelles) around trace amounts of water, placing the hydrophilic moiety towards the water domain and their hydrophobic tails towards the bulk oil [62]. These reverse micelles in bulk oils creates oil–water interfacial nano-environment where hydrophilics (e.g., carbon dots) and amphiphilics (e.g., lipid radicals) can be driven into close contact with each other, thus acting CDs as active antioxidants. To explain the CDs scavenging activity in this scenario, some optical key parameters of CDs were considered. For the prediction of the position of the energy levels of the valence band (E_VB_) and the bottom levels of the conduction band (E_CB_), the Equations (5) and (6) were used [63]:

E_CB_ = χ−E^C^−(E_g_/2) (5)

**Figure 9:**
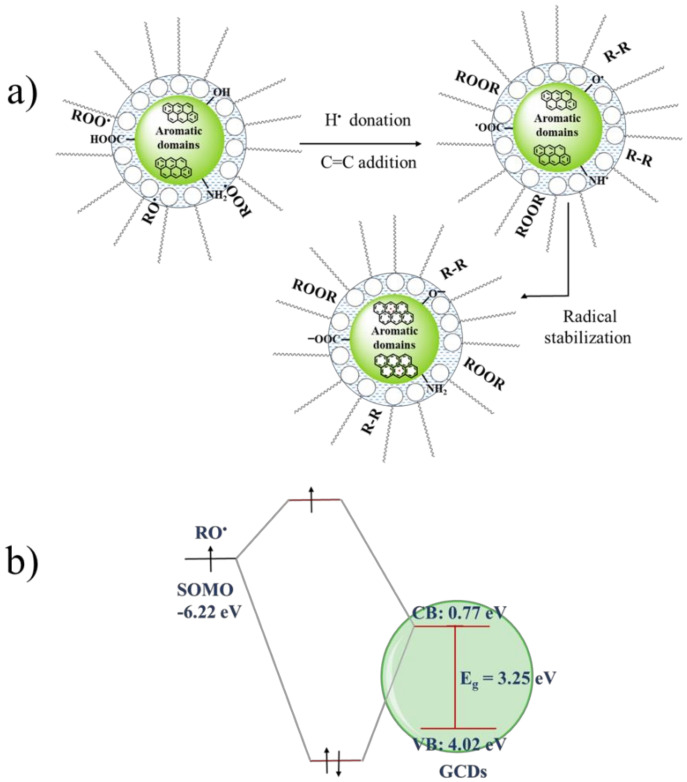
(**a**) Carbon dots antioxidant activity at the interface water-oil mediated by reverse micelles, (**b**) Frontier orbital interactions of singly occupied molecular orbital (SOMO) of the alkoxyl radical (RO^•^) and the conduction band of GCDs.

E_VB_ = E_CB_ + E_g_ (6)

in which E_CB_ and E_VB_ are the potentials of the conduction and valence bands, respectively, E^C^ is the energy of free electrons on the hydrogen scale (~4.5 eV) [64], E_g_ and χ are the band gap and the electronegativity of the CDs, respectively. The χ was expressed as the geometric mean of the absolute electronegativity of the atoms forming the CDs. Taking the GCDs as model, the electronegativity values for C, O, S, N and H atoms were taken as 6.27, 7.54, 6.22, 7.30 and 7.18 eV, respectively [65]. The calculated values of E_CB_ (0.77 eV) and E_VB_ (4.02 eV) were found for GCDs. Three generic alkyl-radical couples, RO^•^/ROH (2.90 eV *vs* NHE), ROO^•^/ROOH (3.50 eV vs NHE), and ROOH/RO^•^ (2.60 eV vs NHE) [65] have been used in the discussion. Considering that the high redox potentials of these radicals make them strongly electrophilic, three possible radical scavenging mechanisms of CDs could be envisaged: (i) addition of the radical to electron rich C=C bonds, (ii) H abstraction from available group donors (-COOH, -SH, -OH) (Figure 9a,c) upon GCDs excitation by light, interaction between the low energy SOMO orbital (single occupied molecular orbital) of the radical and conduction band of the GCDs of higher energy (Figure 9b). After any of the above possibilities, the unpaired electrons may be then delocalized by resonance within the CDs conjugated nano-domains and/or by a rearrangement of chemical bonds. 

## 4. Conclusions

The use of biomass as starting materials for synthetizing CDs is a sustainable way to revalorize industrial wastes. Without further addition of N-doping molecules and using a hydrothermal route, we successfully synthesized CDs from tea wastes (TCDs) and grape pomace (P1CDs and P2CDs) with antioxidant properties and dispersibility both in water and in non-aqueous media. Also, citric acid/glutathione CDs, as representative of CDs obtained from small molecules were produced by hydrothermal synthesis. In aqueous media, the antioxidant properties of all the CDs obtained were determined by DPPH, which compared favorably with those standard compounds such as ascorbic acid and vitamin E and with those of CDs obtained from raw biomaterials. Thus, EC_50_ of reducing power were 50, 75 and 175 μg·mL^−1^ for TCDs, P2CDs and GCDs, respectively. Due to the solubility of theses CDs in non-aqueous media, as a proof of the concept, GCDs and TCDs were used as antioxidant additives in a lubricant base oil. Results render these CDs as potential candidates for applications in the oxidative protection of vegetable-oil based lubes. In this work we demonstrate that CDs obtained from vegetable wastes provide an extraordinary opportunity to study the role played by the intrinsic properties of the starting materials, in particular in terms of their antioxidant activity. The work is a step forward towards the use of CDs as antioxidant additives in lubricant oils as demonstrated by the oxidative stability of a commercial lubricant oil provided by the addition TCDs. In principle, these CDs are expected to be applied in the future not only as green antioxidant additives in bio-lubricants, particularly those based on vegetal oils (or in other products such as cosmetics or food packaging) but also as green anti-friction and anti-wear nano-additives in biodegradable lubes. However, in order to realize the full potential of CDs obtained from biomass resources further long-term studies are certainly needed to clarify the importance of the different origin of the same biomass source, the potential cytotoxicity of the CDs or the synthesis procedures that focus on green chemistry. 

## Figures and Tables

**Table 1:** Carbon dots with antioxidant properties, precursors and applications.

Carbon Precursor	Dopants	Synthesis Method	Methods	Application	EC50 μg mL^−1^	Ref.
Garlic	-	HT 200 °C, 3 h Autoclave	DPPH Fenton	-	80	[13]
Coriander leaves	-	HT 240 °C, 4 h	DPPH	-	15	[14]
Coconut husk	-	HT 200 °C, 3 h. Autoclave	DPPH	-	60	[15]
Coconut shell	-	HT 200 °C, 3 h Autoclave	DPPH	-	25	[16]
Selenocystine	-	HT 60 °C, 24 h	ESRS DEMPO	MDA-MB-231 cells viability	-	[17]
BSA	Ce(NO_3_)_3_·6H_2_O	Bio-mineralization pH 12, 55 °C, 8 h	Methyl Violet	VSMC and 7721 cells viability	-	[18]
Tea waste	Ethylene-diamine	HT 150 °C	Fenton Pyrogallol	-	80 (hydroxyl radical), 24.2 (superoxide radical)	[19]
1,4-phenylene- diamine	Phosphoric acid + Mn(OAc)_2_	HT 220 °C, 10 min Microwave	DPPH Fenton NBT enzymatic	B15F1, HeLa and HEL cells viability	6.55 (DPPH) 6.44 (^•^OH) 4.30 (O_2_^•−^)	[20]
Phloroglucinol + phenol	-	HT 260 °C, 5 h Autoclave	Fenton	rADSC cells viability	-	[21]
Citric acid	Mercapto-ethylamine + sodium selenite	HT 150 °C, 2.5 h	DPPH Fenton DTNB	-	-	[7]
Cumin seeds	-	HT 250 °C, 6 h Autoclave	DPPH	-	1.2	[22]
*Thymus vulgaris* L. essential oil	-	HT 200 °C, 15 h Autoclave	DPPH	-	23.43	[23]
Lutein	Ethylene-diamine	HT 140 °C, 12 h Autoclave	DPPH ROSUP DCFH-DA	NCI-H1299 cells viability	-	[24]
EDTA	Ce(NO_3_)_3_·6H_2_O Pr(NO_3_)_3_·6H_2_O	HT 200 °C, 8 h	Fenton ESRS	MEF and PATU 8988 cells viability	-	[25]

DEMPO = 5,5′-dimethylpyrroline N-oxide; DCFH-DA = 2′,7′-dichlorofluorescein diacetate; DPPH = 1,1-Diphenyl-2 trinitrophenylhydrazine; DTNB = 5,5′-dithiobis(2-nitrobenzoic acid); EDTA = ethylenediaminetetraacetic acid; ESRS = Electron Spin Resonance Spectrometry; HT= hydrothermal NBT = nitro blue tetrazolium; ROSUP = (ROS reagent, as a positive control for ROS level for cells).

**Table 2:** Optical and electronic properties of the CDs.

Carbon Dots	Bandgap, eV	Urbach Energy, meV	λ_exc_, nm	λ_em_, nm	Δλ, nm	QY, %
GCDs	3.25	337	350	418	-	55.7
TCDs	3.50	626	350	430	Δλ_em_ = 22	4.50
P1CDs	3.90	583	350	423	Δλ_em_ = 5	2.76
P2CDs	3.10	486	318	440	Δλ_ex_ = 32 Δλ_em_ = 10	0.65

**Table 3:** Comparison of EC_50_ values (DPPH assay) for the CDs studied with those of some reported materials.

Antioxidant Material	Synthesis Method	EC_50_ μg mL^−1^	Ref.
Coconut husk-CDs	HT 200 °C, 3 h. Autoclave	60	[15]
Coconut Shell-CDs	HT 200 °C, 3 h Autoclave	25	[16]
Ascorbic acid	Pure compound	15.0	[49]
Vitamin E	Pure compound	13.58	[50]
HcEd *	Methanol extraction	16.7	[51]
Essential oil of *Teucrium orientale subsp. taylori*	Hydrodistillation extraction	121.60	[52]
Ginger essential oil	Hydrodistillation extraction	11,680	[53]
TCDs	HT 200 °C, 6 h Oven	50	This work
P2CDs	HT 180 °C, 4 h Oven	75	
GCDs	HT 180 °C, 5 h Oven	175	

* Essential oil extracted in methanol from *Hyptis crenata* Pohl ex Bentham.

## Supplementary Materials

The following are available online at https://www.mdpi.com/2076-3921/9/11/1147/s1, Figure S1: HRTEM image of P2CDs showing uniform dispersity of carbon dots (small dark spots) at 100 nm resolution; Figure S2. Urbach energy estimation for GCDs; Figure S3. Urbach energy estimation for TCDs; Figure S4. Urbach energy estimation for P1CDs; Figure S5. Urbach energy estimation for P2CDs; Figure S6. Excitation and emission spectra of the synthetized carbon dots. The spectra were vertically shifted for the sake of clarity; Table S1. Some key tribological properties of the mineal ISO 68 oil (Repsol data).
