# Supplementary material for: Synthesis and Characterization of Green Carbon Dots for Scavenging Radical Oxygen Species in Aqueous and Oil Samples
Source: Antioxidants (Basel). 2020 Nov 19;9(11):1147. doi: 10.3390/antiox9111147 (PMC7699408; doi:10.3390/antiox9111147)
Supplement: Supplementary file 1 [file antioxidants-09-01147-s001.pdf]

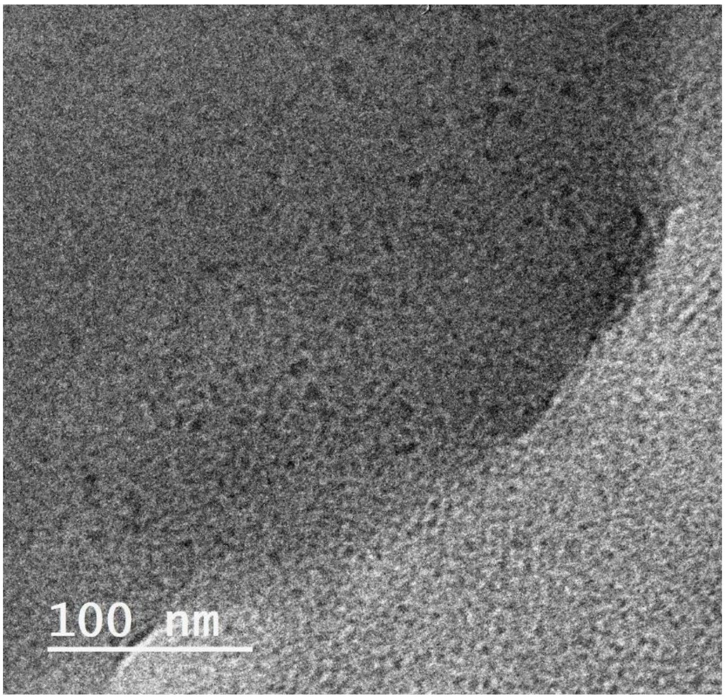

Figure 1. HRTEM image of P2CDs showing uniform dispersity of carbon dots (small dark spots) at 100 nm resolution.

Estimation of Urbach energy (E_u).

Optical absorption in semiconductors, near band edges, promotes an electron from the top of the valence band into the bottom of the conduction band across the energy band gap. During this transition process, if these electrons encounter disorder or defects (the band edges at VB and CB are not well-defined cut-off energies), and the density of states falls quickly into the bandgap, the absorption coefficient $\alpha(h\nu)$ tails off depending exponentially on hν. Such dependence is called the Urbach rule given by the equation:

$$\alpha(h\nu) = \alpha_0 \exp(h\nu/E_u) \quad (1)$$

where α_o is a material-dependent constant, hν is the photon energy and E_u is the Urbach energy (also called Urbach width). The Urbach energy was estimated from the reciprocal of slopes derived from fits of linear portion in the lower photon energy region of ln $\alpha(h\nu)$ vs. hν plots.

The Urbarch energy plots for GCDs, TCDs, P1CDs and P2CDs are depicted in Figures 2S to 5S. The inset shows the linear adjustment in each case.

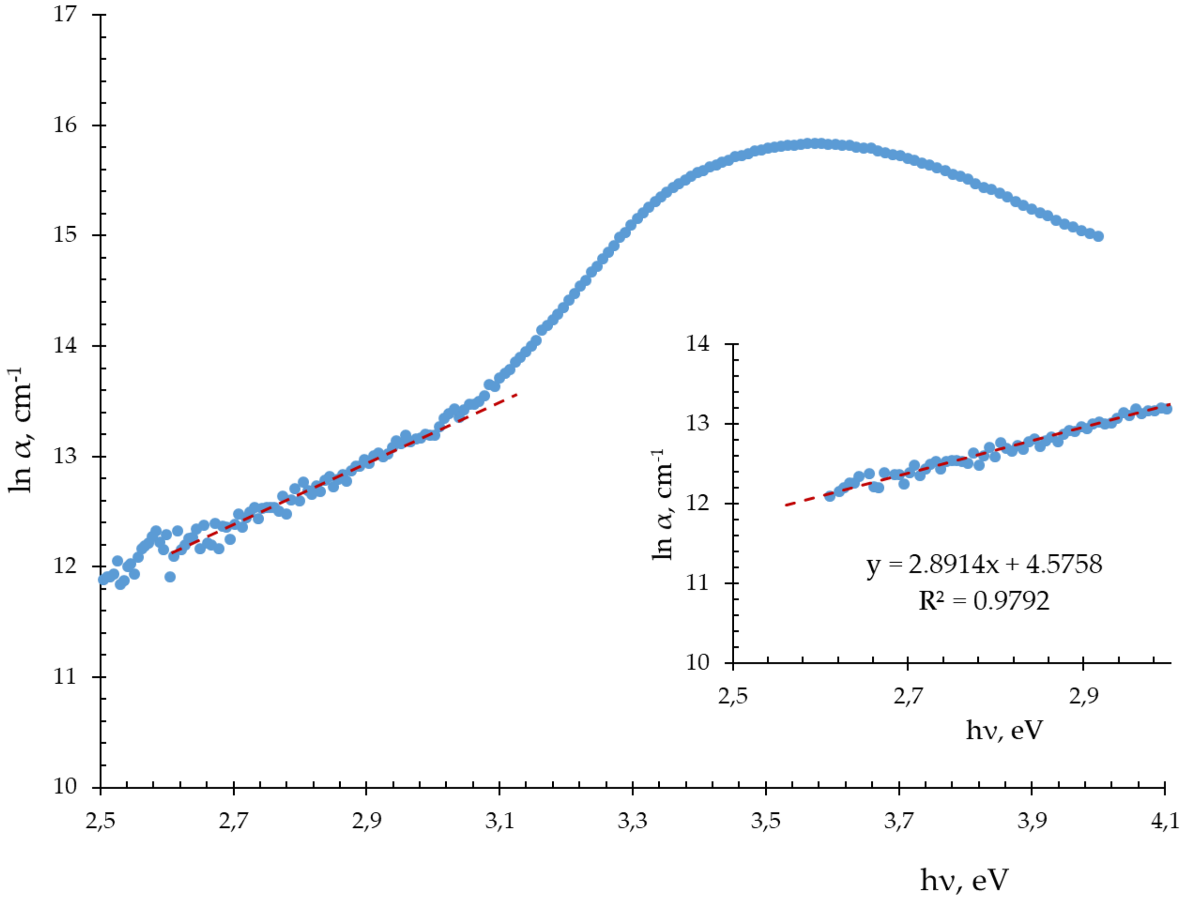

Figure 2. Urbach energy estimation for GCDs.

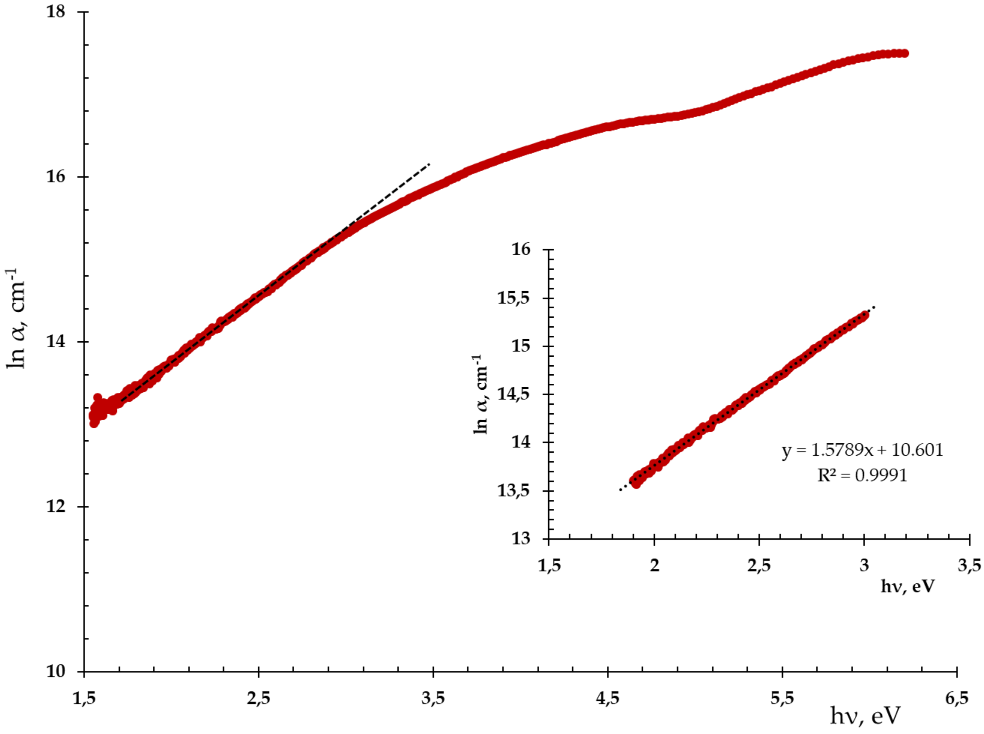

Figure 3. Urbach energy estimation for TCDs.

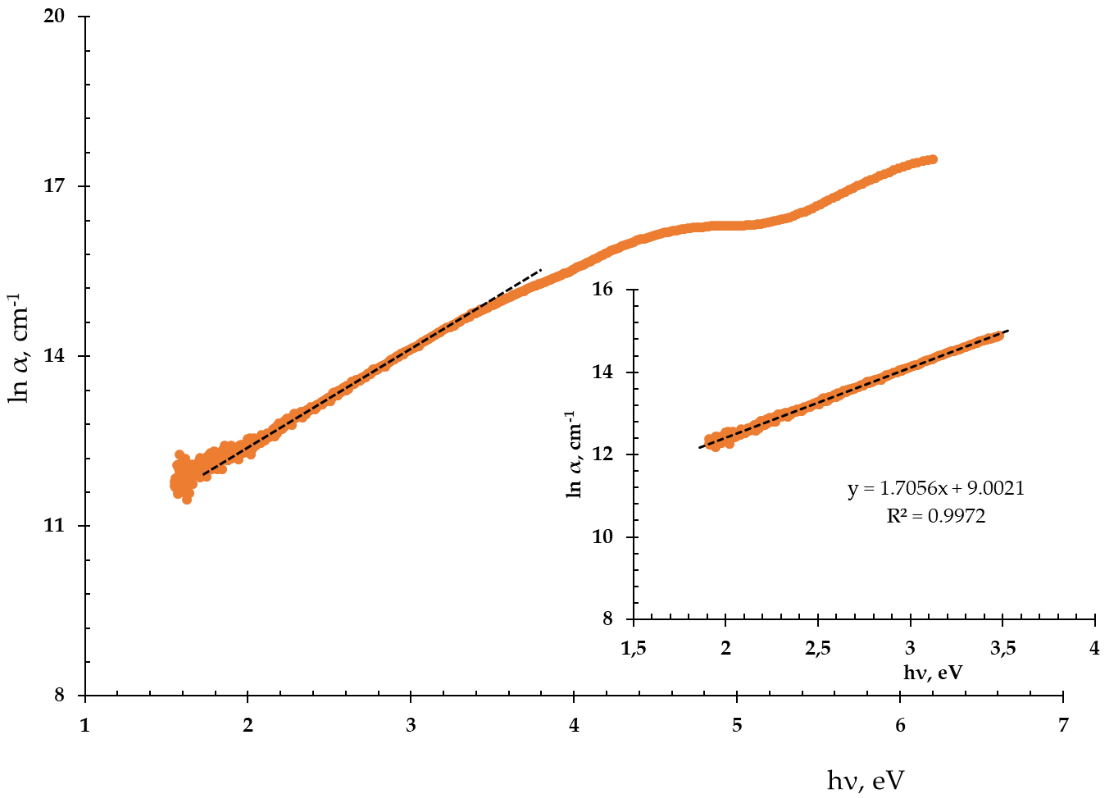

Figure 4. Urbach energy estimation for P1CDs.

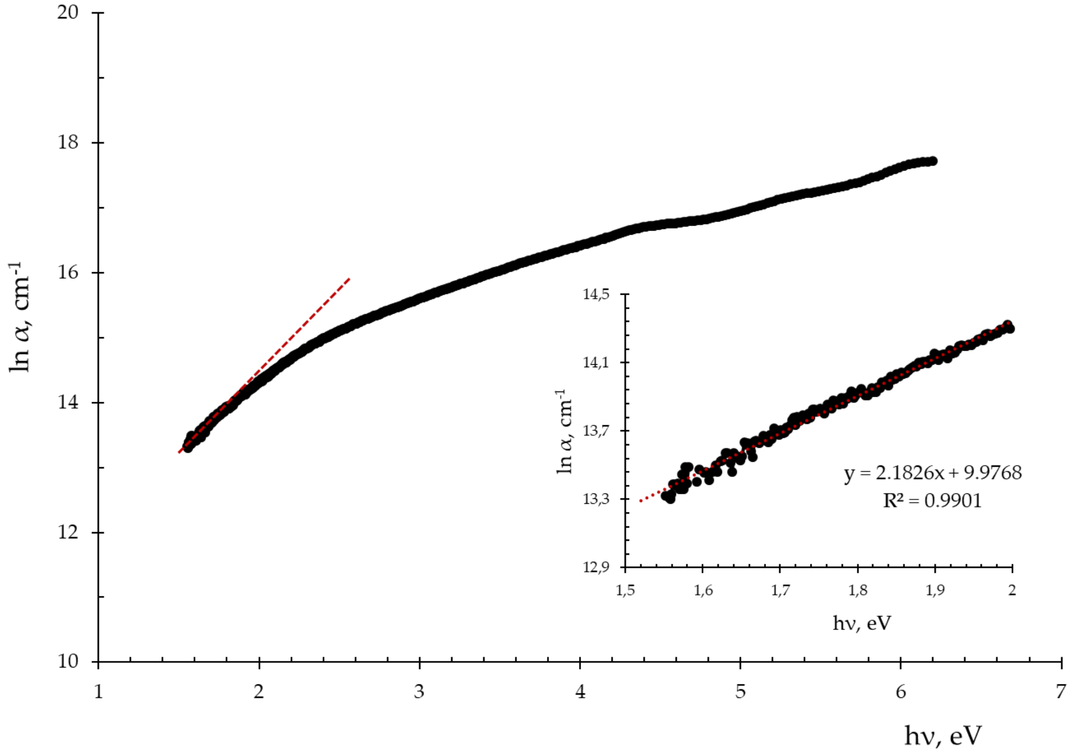

Figure 5. Urbach energy estimation for P2CDs.

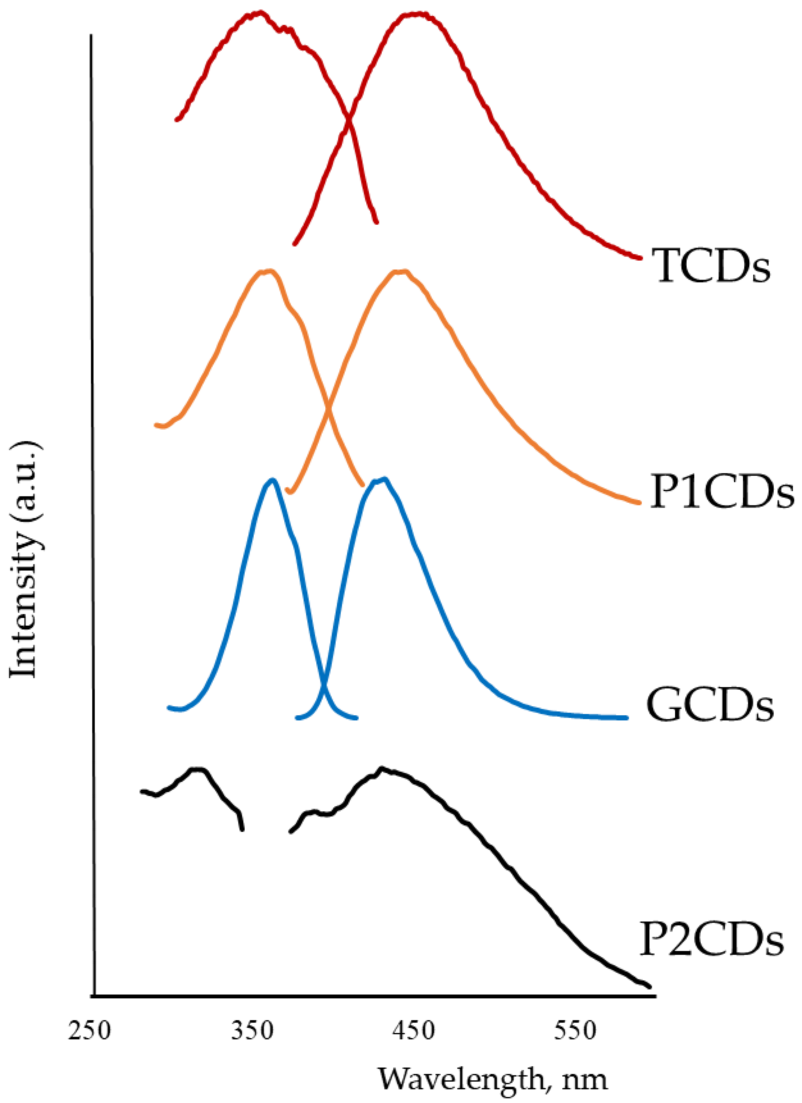

Figure 6. Excitation and emission spectra of the synthetized carbon dots. The spectra were vertically shifted for the sake of clarity.

Table 1. Some key tribological properties of the mineal ISO 68 oil [Repsol data].

Property	Method	ISO 68
Viscosity at 100 °C	ASTM D 445	8.6 cSt
Viscosity at 40 °C	ASTM D 445	68 cSt
Density at 15 °C	--	0.88 g·cm^{-3}
Total acidity number (TAN)	ASTM D 664	0.38 KOH·g^{-1}
Flash point	ASTM D 92	235 °C
Pour point	ASTM D 97	-24 °C
